# Laparoscopic Heller-Dor Is an Effective Treatment for Esophageal-Gastric Junction Outflow Obstruction

**DOI:** 10.1007/s11605-021-05021-1

**Published:** 2021-05-06

**Authors:** Renato Salvador, Luca Provenzano, Giulia Nezi, Giovanni Capovilla, Loredana Nicoletti, Elisa Sefora Pierobon, Lucia Moletta, Michele Valmasoni, Stefano Merigliano, Mario Costantini

**Affiliations:** Department of Surgical, Oncological and Gastroenterological Sciences, University of Padova, School of Medicine, Clinica Chirurgica 3, Azienda Ospedale Università di Padova, Padova, Italy

**Keywords:** Heller-Dor, EGJOO, High-resolution manometry

## Abstract

**Background:**

The treatment of esophagogastric junction outflow obstruction (EGJOO) currently mirrors that of achalasia, but this is based on only a few studies on small case series. The aim of this prospective, controlled study was to assess the outcome of laparoscopic Heller-Dor (LHD) in patients with EGJOO, as compared with patients with esophageal achalasia.

**Materials and Methods:**

Between 2016 and 2019, patients with manometric diagnosis of idiopathic EGJOO and patients with radiological stage I achalasia, both treated with LHD, were compared. The achalasia group was further analyzed by subgrouping the patients based on the manometric pattern. Treatment failure was defined as the persistence or reoccurrence of an Eckardt score > 3 or the need for retreatment.

**Results:**

During the study period, 150 patients were enrolled: 25 patients had EGJOO and 125 had radiological stage I achalasia (25 pattern I, 74 pattern II, and 26 pattern III). The median follow-up was 24 months (IQR: 34–16). Treatment was successful in 96% of patients in the EGJOO group and in 96% of achalasia patients with pattern I, 98.7% in those with pattern II, and 96.2% of those with pattern III (*p*=0.50). High-resolution manometry showed a reduction in the LES resting pressure and integrated relaxation pressure for all patients in all 4 groups (*p*<0.001).

**Conclusion:**

This is the first comparative study based on prospective data collection to assess the outcome of LHD in patients with EGJOO. LHD emerged as an effective treatment for EGJOO, with an excellent success rate, comparable with the procedure’s efficacy in treating early-stage achalasia.

## Background

Esophago-gastric junction outflow obstruction (EGJOO) is a relatively new clinical entity revealed by high-resolution manometry (HRM). According to the Chicago Classification, the disorder is due to a poorly relaxing lower esophageal sphincter (LES), with integrated relaxation pressure (IRP) >15 mmHg, and a preserved esophageal peristalsis.^1^

This manometric diagnosis can have two different etiologies, i.e., idiopathic or secondary EGJOO. The latter may be caused by any mechanical obstruction of the esophagus, such as esophageal strictures, eosinophilic esophagitis, giant hiatal hernia, prior fundoplication or bariatric surgery, or malignancy.^2^ Patients with no such anatomical obstructions are diagnosed with idiopathic EGJOO. The main clinical manifestations of idiopathic EGJOO are dysphagia, chest pain, and regurgitation.^3^ For the time being, there are no published guidelines on the management and treatment of this recently identified motility disorder. Many authors have suggested using the same treatment for EGJOO as for achalasia, but this recommendation is based on only a few studies with small case series. Oral calcium-channel blockers are the initial treatment of choice, but achieve low rates of symptom relief of around 35%.^4^

The aim of this prospective, controlled study was to examine the outcome of laparoscopic Heller-Dor (LHD) in patients with idiopathic EGJOO, as compared with that of patients with radiological grade I esophageal achalasia.

## Materials and Methods

### EGJOO Padova Protocol

All patients with a diagnosis of idiopathic EGJOO (i.e., not postoperative or associated to hiatal hernia or esophageal stenosis) who underwent HRM (Manoview, Medtronic Minnesota, USA) at the Department of Surgical, Oncological, and Gastroenterological Sciences, University of Padova (Italy) from 2016 to 2019, and referred for dysphagia or food regurgitation, were prospectively collected. Patients with a manometric diagnosis of idiopathic EGJOO after being referred for chest pain, heartburn, or other extra-esophageal symptoms were ruled out. EGJOO was diagnosed on the grounds of accepted esophageal motility characteristics, i.e., IRP >15 mmHg with a preserved esophageal peristalsis, according to the Chicago Classification v3.0 criteria.^1^

**Fig. 1:**
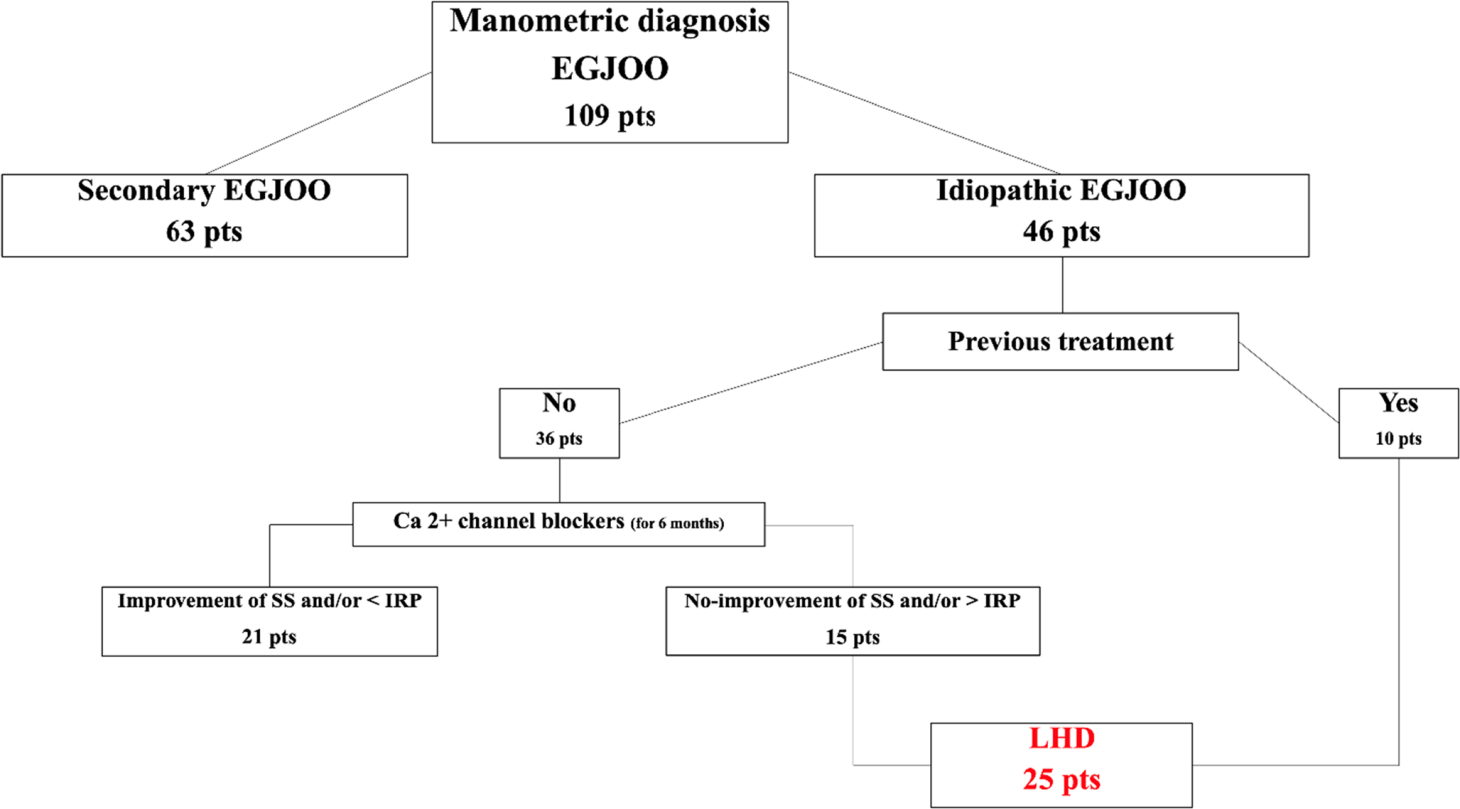
Flow-chart for selection of patients with a manometric diagnosis of EGJOO complaining of dysphagia and/or food-regurgitation

Naïve EGJOO patients (i.e., patients who did not have a previous endoscopic treatment) were first given medical treatment with Ca2+ channel blockers for 6 months. Then patients were “restaged” on the strength of repeat HRM and the symptom score (SS). Patients whose SS had not improved and/or whose IRP had not decreased were scheduled for LHD (Fig. 1). Patients referred to our center with a previous diagnosis of EGJOO who had already been treated with botox injections, pneumatic dilations, or Ca2+ channel blockers were scheduled upfront for LHD.

Patients with EGJOO who underwent LHD were compared with patients with radiological stage I achalasia (i.e., undergoing the same procedure during the same period. The achalasia group was subgrouped for the purposes of our analyses based on patients’ manometric patterns I, II, or III.

Patients’ demographics, clinical data, and symptoms were prospectively collected with a dedicated database adopted at our center. Patients’ demographics, clinical data, and symptoms were collected with a dedicated questionnaire adopted at our center. To evaluate the symptoms score (SS), we used the Eckardt score.^5^

Before LHD, all patients had a barium swallow X-ray to assess the diameter of the esophagus,^6^ and endoscopy to rule out cardia malignancies, eosinophilic esophagitis, and/or other esophageal diseases.

The surgical technique for LHD has been described in detail elsewhere^7^ and was performed in the same way by two expert surgeons (RS, MC). Briefly, a myotomy 7–8 cm long was performed after dissecting only the anterior wall of the esophagus, extending the myotomy 1.5–2 cm on the gastric side. During the procedure, a 30-mm Rigiflex balloon was placed inside the esophageal lumen at the cardia level, using an endoscopically positioned guidewire. The balloon was then gently inflated with air and deflated while the muscle fibers were being cut. A partial anterior fundoplication according to the technique described by Dor was performed and sutured to the edges of the myotomy with three stitches on each side. The more proximal suture included the homolateral pillar of the hiatus to keep the fundoplication high around the esophagus.

**Fig. 2:**
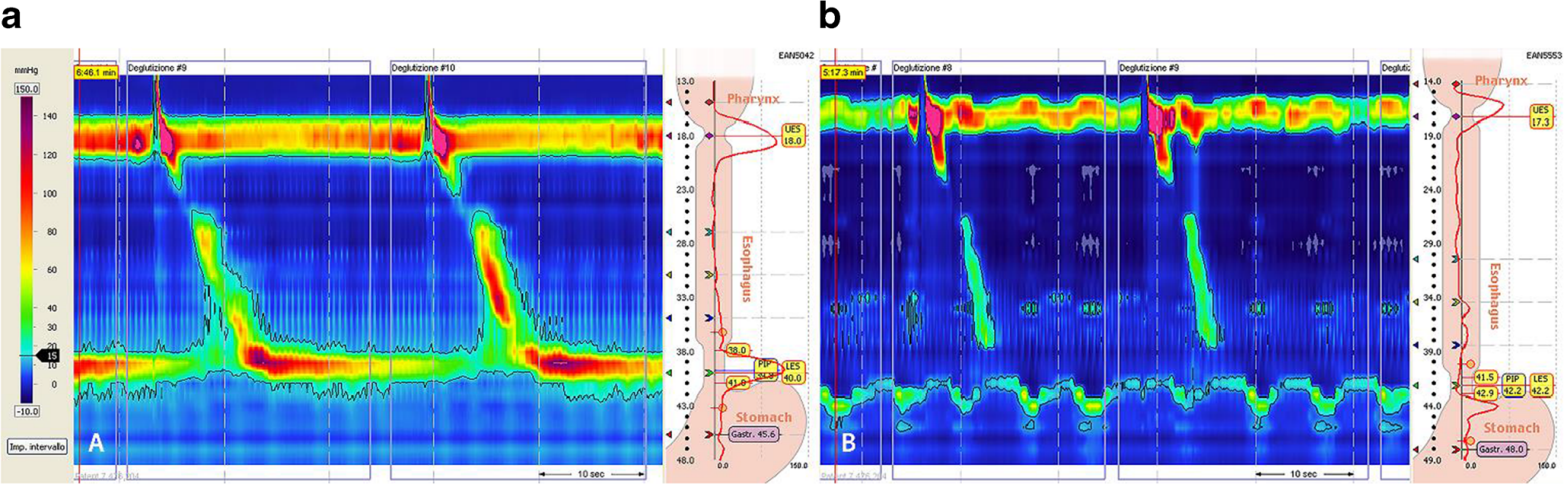
High-resolution manometry pictures of EGJOO: a preoperatively and b postoperatively

The postoperative follow-up included examining patients 1, 6, and 12 months after surgery, and every 2 years thereafter, using the same questionnaire that was administered preoperatively. Barium swallow X-ray was required 1 month after LHD and in the event of recurrent symptoms. HRM was performed 6 months after LHD (Fig. 2), together with 24-h pH monitoring to check for any abnormal acid exposure of the distal esophagus. Endoscopy was recommended 12 months after LHD, and then every 2 years thereafter,^8^ to identify and control any complications of gastroesophageal reflux and rule out any (albeit rare) neoplastic degeneration (Table 1).

**Table 1:** Follow-up procedures and timing

Procedure	1 month	6 months	12 months	Every 2 years
Symptom questionnaire (Eckardt score)	■	■	■	■
Barium swallow	■			
High-resolution manometry		■		
24-h pH monitoring		■		
Endoscopy			■	■

Treatment failure was defined as the persistence or reoccurrence of an Eckardt score > 3 or the need for retreatment.^9^, ^10^

The study was approved by the institutional review board at Padova General Hospital and by the Research Committee of the Department of Surgical, Oncological, and Gastroenterological Sciences at Padova University. Research is being reported in line with the STROCCS criteria.^11^ This study was registered in the Research Registry (research registry 6562).

### Statistical analysis

Continuous data were expressed as medians and interquartile ranges (IQR), and categorical data as numbers and percentages. Continuous and categorical data were compared between the four study groups using the Kruskal-Wallis test and Fisher’s test, respectively. All tests were two-sided and a *p* value of less than 0.05 was considered statistically significant.

## Results

During the study period, we examined 109 patients referred for dysphagia and/or food-regurgitation who had EGJOO diagnosed on HRM. Secondary EGJOO (i.e., associated with prior fundoplication, hiatal hernia, esophageal stenosis, or other causes) was identified in 63 patients, who were therefore excluded from the study.

Idiopathic EGJOO was diagnosed in 46 patients. Of the 36 previously untreated, 15 patients experienced no symptom improvement or reduction in IRP after 6 months of therapy with Ca2+ blockers. These patients were offered LHD and entered the study together with the 10 patients who had previously been treated endoscopically. The study population thus included 25 patients (M:F=15:10). Figure 1 shows the flowchart for the selection of our study population.

Our control population included 125 patients with radiological stage I achalasia. According to the Chicago Classification v3.0, 25 patients were classified as having pattern I, 74 had pattern II, and 26 had pattern III.

Table 2 shows the demographic and clinical parameters of the study population and control group. A history of endoscopic treatments (pneumatic dilations, botox injections, or both) was more common in the EGJOO group (40%) than in any of the 3 achalasia subgroups (16% of patients with pattern I, 12.2% of those with pattern II, and 7.7% of those with pattern III) (*p*=0.01).

**Table 2:** Pre-operative demographic and clinical findings in the four groups of patients

	EGJOO	Achalasia pattern I	Achalasia pattern II	Achalasia pattern III	*p* value
Patients (*n*)	25	25	74	26	
Age (years)*	65 (50–74)	44 (30–60)	48 (33–61)	56 (39–66)	<0.01
Sex (M: F)	15:10	12:13	30:44	19:7	0.007
Symptom duration (months)*	34 (21–60)	24 (12–84)	24 (23–36)	30 (23–60)	0.44
Symptom score (Eckardt score)*	5 (3–6)	8 (6–9)	7 (6–8)	7 (4–9)	<0.01
Previous endoscopic treatment^	10 (40%)	4 (16%)	9 (12.2%)	2 (7.7%)	0.01
Esophageal diameter (mm)*	21 (20–29)	30 (24–30)	27 (23–30)	25 (20–26)	0.11
LES resting pressure (mmHg)*	42 (27–50)	43 (30–53)	50 (41–60)	49 (35–65)	0.051
IRP (mmHg)*	20 (19–23)	27 (22–29)	35 (26–46)	26 (20–39)	<0.001

Data are shown as * median (IQR), ^ number of patients (%)

Abbreviations: *EGJOO* esophagogastric junction outflow obstruction, *LES* lower esophageal sphincter, *IRP* integrated relaxation pressure

The EGJOO group had a lower preoperative IRP (20 mmHg, IQR: 19–23) than patients with achalasia (pattern I: 27 mmHg, IQR: 22–29; pattern II: 35mmHg, IQR: 26–46; pattern III: 26 mmHg, IQR: 20–39) (*p*<0.001).

The surgical procedure was completed laparoscopically in all patients. Mortality was nil. Intraoperative perforations were detected in two patients, one with EGJOO and one with pattern II achalasia. Both of these mucosal tears were repaired during the LHD procedure and were without further consequences. Gastrografin swallow X-ray performed on the first postoperative day after LHD revealed no mucosal tears in any of the patients.

The median follow-up was 24 months (IQR: 34–16). All patients had a lower SS after LHD; the median of preoperative SS was 7 (IQR: 5–8) while the postoperative was 0 (IQR: 0–1) (*p*<0.001). During the follow-up, the outcome was considered *satisfactory* in 96% of patients in the EGJOO group, and in 96% in the achalasia pattern I subgroup, 98.7% in the achalasia pattern II subgroup, and 96.2% in the achalasia pattern III subgroup (*p*=0.50). There were four treatment failures in all, one in each group or subgroup. All patients whose surgery failed subsequently underwent one or more endoscopic pneumatic dilation treatments, obtaining an improvement in their symptom scores.

Postoperative HRM showed a reduction in the LES resting pressure and IRP in all patients in all 4 groups/subgroups (*p*<0.001). The median postoperative IRP was similar in all groups as well: 9 mmHg (IQR: 8–12) in the EGJOO group, 11 mmHg (IQR: 10–12) in the pattern I subgroup, 9 mmHg (IQR: 6–12) in the pattern II subgroup, and 12 mmHg (IQR: 7–14) in the pattern III subgroup (*p*=0.51). In addition, 24-h pH monitoring showed comparable proportions of postoperative abnormal acid exposure in all groups/subgroups, affecting 14.3% of EGJOO patients, 8.3% of achalasia pattern I patients, 8.9% of achalasia pattern II patients, and 10% of achalasia pattern III patients (*p*=0.49) (Table 3).

**Table 3:** Post-operative parameters

	EGJOO	Achalasia pattern I	Achalasia pattern II	Achalasia pattern III	*p* value
Symptom score (Eckardt score)*	0 (0–1)	0 (0–1)	0 (0–1)	0 (0–0)	0.20
LES resting pressure (mmHg)*	19 (15–22)	17 (16–18)	18 (13–23)	17 (16–18)	0.41
IRP (mmHg)*	9 (8–12)	11 (10–12)	9 (6–12)	12 (7–14)	0.51
Positive outcome	96%	96%	98.7%	96.2%	0.50
Abnormal 24-h pH-monitoring	14.3%	8.3%	8.9%	10%	0.49

Data are shown as * median (IQR), ^ number of patients (%)

Abbreviations: *EGJOO* esophagogastric junction outflow obstruction, LES lower esophageal sphincter, *IRP* integrated relaxation pressure

## Discussion

The advent and now widespread diffusion of HRM prompted a new classification of esophageal motility disorders, the Chicago Classification, which is currently in its 3^rd^ version.^1^ This classification is based on a dichotomous hierarchical tree where the main distinction is between a normal and an abnormal LES relaxation (IRP <15 mmHg or >15 mmHg, respectively). Combined with the absence of any peristalsis, an abnormal LES relaxation is the main characteristic of achalasia, in its 3 different manometric patterns. A poor LES relaxation associated with a preserved peristalsis (and therefore lacking one of the defining features of achalasia) can still interfere with bolus transit to the stomach and cause dysphagia. This major motility disorder is characterized by an elevated LES relaxation pressure (IRP >15 mmHg) associated with an intact peristalsis.

EGJOO is a relatively new manometric diagnostic entity, and its etiology may be idiopathic or secondary to mechanical obstructions, such as esophageal strictures, eosinophilic esophagitis, giant hiatal hernia, prior fundoplication or bariatric surgery, or malignancy.^2^ Patients without any such anatomical obstruction are diagnosed with idiopathic EGJOO. The pathophysiology of “idiopathic” EGJOO is unclear, but most authors see it as a precursor or variant of achalasia, as clearly suggested by its description in the Chicago Classification as “incompletely expressed achalasia.” This theory is only supported, however, by small case series in which some EGJOO patients developed achalasia after treatment.^12^

EGJOO is a relatively uncommon condition^13^; as in achalasia, the main clinical manifestations of idiopathic EGJOO are dysphagia, chest pain and regurgitation,^3^ and treatments that aim to relieve the idiopathic obstruction (i.e., to improve esophago-gastric junction relaxation) are the most effective. So far, the management of this novel motility disorder has been based on a handful of studies on small case series, in the light of experience gained with achalasia.

Symptoms in patients with EGJOO do not always require treatment and their final outcome varies, so deciding which patients need intervention is a key challenge in these patients’ management.^3^, ^13^ Our study confirmed this point of view because we observed that patients who responded to medical management did not present any radiological or manometric difference compared to patients who had not a clinical improvement with Ca2+ channel blockers. Some authors suggest that 44–52% of patients diagnosed with EGJOO improve without any pharmacological or surgical therapy.^3^, ^14^, ^15^ The present study enrolled only idiopathic EGJOO patients with symptoms of dysphagia and food regurgitation who did not respond to medical treatment.

Treatment options for idiopathic EGJOO described in the literature include medical, endoscopic and surgical approaches. Medical treatments such as Ca2+ channel blockers and long-acting nitrates have been used, but the few data reported by Perez-Fernandez et al. and other authors point to low clinical response rates, involving only about 50% of patients with EGJOO.^14^ Porter et al. reported on the treatment of 36 EGJOO patients with botox injections. Their survival analysis showed that persistent symptom relief from a single botox injection at 2 years was just 16.8%.^16^ Some authors also found that some patients treated with botox injections progressed to type 3 achalasia within 20 months of this treatment.^2^

Given that idiopathic EGJOO appears to be achalasia’s precursor, Clayton et al. treated patients with pneumatic dilations. They found this a safe and effective initial treatment for idiopathic EGJOO in patients with dysphagia and abnormal esophageal emptying. The outcome was positive in 67% of patients (22/33) treated with a single dilation using a 30-mm balloon, and in 79% of patients retreated with a 35-mm balloon, after a median follow-up of around 2 years.^4^

In an international multicenter study, Khashab et al. assessed the clinical outcomes of peroral endoscopic myotomy (POEM) in patients with disorders other than achalasia, i.e., EGJOO, diffuse esophageal spasm, and jackhammer esophagus. They reported a positive outcome in 93% of EGJOO patients after POEM. The main limitation of this study is the short follow-up, which was only about 6 months.^17^ These positive findings were replicated by Okeke et al., however, who treated three patients with POEM, obtaining a 100% success rate after long-term follow-up.^18^

The aim of the present study was to ascertain whether LHD has a role in the treatment of patients with idiopathic EGJOO, a hypothesis that—in our opinion—is confirmed by our data. For now, there are no published studies dedicated to examining the role of LHD for this recently identified clinical entity. In a recent review, Clayton et al. sought to collect all the cohorts of EGJOO patients treated with Heller myotomy: they found only two case series concerning a total of four patients treated, and all four of them experienced lasting symptom relief.^3^, ^4^, ^19^ Lin et al. published a case report on a robot-assisted extended esophageal myotomy and Belsey-Mark IV fundoplication performed in a patient diagnosed with EGJOO who had suffered from severe headache using calcium channel blockers and nitrites for pain control. At one-year follow-up, the patient’s chest pain and dysphagia had improved.^20^

Our study confirmed the value of surgical myotomy for EGJOO, showing that patients who underwent LHD achieved symptom relief in 96% of cases. This figure is comparable with the results achieved at our center using the same procedure in patients with esophageal achalasia.^21^–^23^ Judging from our results, moreover, LHD in EGJOO was also comparable with LHD for esophageal achalasia in terms of its influence on any onset of postoperative gastroesophageal reflux.

This is the first comparative study based on prospective data collection to assess the outcome of LHD in patients with EGJOO. It has some intrinsic limitations, however, that need to be mentioned. The main limitation concerns the number of patients considered was small (though ours is the second-largest cohort of patients treated for EGJOO). A second limitation may be the exclusion of EGJOO who were referred for chest pain only (without dysphagia or food regurgitation).

## Conclusion

We are nonetheless convinced that LHD could be an effective treatment for EGJOO, comparable with what the same procedure can achieve in early-stage achalasia. Further studies with longer follow-up and a larger cohort of EGJOO patients need to confirm our results. LHD should be offered to EGJOO patients in dedicated center only, with great experience with this operation and esophageal diseases. Additional studies might better pinpoint EGJOO patients and help us to establish whether this condition is a variant or an early stage of achalasia.
